# The long-term physical, emotional and psychosexual outcomes related to anal incontinence after severe perineal trauma at childbirth

**DOI:** 10.18332/ejm/93544

**Published:** 2018-08-01

**Authors:** Angeliki Antonakou

**Affiliations:** 1 Midwifery Department, Alexander Technological Educational Institute of Thessaloniki, Greece

**Keywords:** perineum, trauma, childbirth, anal incontinence, psychosexual morbidity, anxiety

Obstetric anal sphincter injuries (OASIS), or third-degree and fourth-degree perineal tears, represent a serious complication at childbirth. The rate of OASIS for primiparous women in the United Kingdom has been reported to have increased from 1.8 to 5.9% over a twelveyear period^1^. In women who suffered OASIS at first birth, the incidence of a repeat OASIS has been shown to be 8.4% in hospital-based studies^2^ and 7.2% in population-based studies^3^ in the United Kingdom. In other countries, the rate of repeat OASIS in hospital-based and population-based studies has been reported to range widely from 2.0 to 7.2%^4,5^ and from 5.6 to 7.1%^6,7^, respectively.

Even though there are well defined risk factors for the occurrence of OASIS, such as nulliparity and instrument assistance at birth, the combination of these risk factors in different scoring systems does not allow the accurate prediction of OASIS^8^. Also, there have been only few interventions described that might reduce the risk of OASIS, such as ‘hands-on’ perineal protection at crowning^9-11^, the use of warm compressions during the second stage of labour and perineal massage during pregnancy^12^.

Using a 5% average rate of OASIS means that 1/20 first time mothers will suffer severe trauma of their perineum at vaginal birth that cannot be predicted, while there are few available interventions, of yet unclear benefit, to reduce its occurrence at childbirth. Furthermore, the associated morbidity is significant to these women as OASIS is a prominent risk factor for the development of anal incontinence (AI)^13^, which is defined as the involuntary leakage of flatus or/and faeces^14^. Women who have sustained OASIS at birth have a two-fold to three-fold greater risk of developing subsequent AI^13^, with rates of AI ranging between 7 to 61%^15^. It has also been reported that 20 to 40% of women still described symptoms of AI at twelve months after birth^16^, with significantly increased risks of AI even after 15 years from childbirth^17^. In the case of long-term AI, there are additional factors that have been reported to aggravate the severity of bowel symptoms, such as age and menopause^17^.

Women who have sustained OASIS at first birth and have a subsequent second pregnancy are exposed to the risk of a repeat OASIS that has been well quantified in the literature^2,3^, and to the potential risk of developing AI that is still to be determined with ongoing research^18^. Even if a repeat OASIS does not occur in the second childbirth, symptoms of AI might still present as a consequence of cumulative pudendal neuropathy, prolonged second labour or even an instrumental delivery at second birth^18^. On the other hand, there are recent reports that the risk of developing long term AI is not associated with the second delivery but only with the severity of OASIS at first childbirth^19^. Moreover, an elective cesarean delivery at second pregnancy has been quoted not to be protective of AI even in cases of a fourthdegree perineal tear at first birth^19^.

Most studies in the literature have focused on the general impact of AI on the quality of life, with only few studies focusing on the specific emotional and psychological consequences of AI following OASIS at childbirth. Women suffering with AI who sustained OASIS at birth have higher rates of negative body image, sexual dysfunction and increased levels of anxiety and depression^20-22^. It has been described that these women feel embarrassed and ashamed^21^. Women with AI resulting from OASIS have been described recently to have a previously unrecognised syndrome called the ‘OASIS syndrome’ that represents a complex of particular social, emotional and psychosexual suffering^22^. Women with this ‘OASIS syndrome’ suffer the physical symptoms of AI in silence and feel too embarrassed to seek help as they feel stigmatised. They feel unclean and develop rituals of repetitive cleaning on a daily basis; they suffer guilt, fear, social isolation and loss of confidence. They suffer significant sexual morbidity and marital distress, with 1/8 never resuming intercourse and 1/12 experiencing a failed partnership or marriage^22^. Some of these women feel that even their maternal role is compromised by their condition^22^.

Despite the known association between OASIS and AI after birth, there are reports that only 1/5 with OASIS are being asked by their health care practitioners if they have bowel symptoms after childbirth^23^. This finding reflects the fact that health professionals either misunderstand or lack the interest and knowledge of how to investigate and support these women^22^. Since the emotional, social and psychosexual adverse effects of AI after OASIS represent a recognisable syndrome, there should be greater awareness by midwives to detect it early and to promptly refer these women in the postpartum period for further assessment, support and management^22,24,25^. In the United Kingdom there is currently no nationally agreed care pathway for women with AI following OASIS or any appropriate questionnaire of bowel symptoms that could assist midwives in their role of identifying these women^22^.

At the moment, midwives should inform women that the bowel function in the postnatal period is an area that warrants serious attention and that being anally incontinent is not a normal consequence of having a baby, and therefore they should be empowered to seek help and support. This would be in line with the role of healthcare practitioners in improving patient safety in health systems worldwide as advocated in the World Health Organization fundamental principles^26^. Moreover, in the ongoing research on patient safety in the context of maternity care with regard to the occurrence of anorectal symptoms after childbirth, the key concepts that need to be addressed involve the potential lack of communication between health practitioners and these women, as well as the management of this stressful and debilitating harmful event once it has occurred^27^.

## CONFLICTS OF INTEREST

Author has completed and submitted the ICMJE Form for Disclosure of Potential Conflicts of Interest and none was reported.
